# Robotic trachelectomy with sentinel lymph node biopsy for cervical cancer: a prospective study investigating minimally invasive radicality

**DOI:** 10.1007/s10147-025-02718-0

**Published:** 2025-03-05

**Authors:** Hiroaki Kobayashi, Shintaro Yanazume, Masaki Kamio, Shinichi Togami, Takashi Ushiwaka

**Affiliations:** Department of Obstetrics and Gynecology, Faculty of Medicine, Kagoshima University, 8-35-1 Sakuragaoka, Kagoshima, 890-8520 Japan

**Keywords:** Cervical cancer, Fertility-sparing, Robotic surgery, Radical trachelectomy, Sentinel lymph node

## Abstract

**Objective:**

The importance of minimally invasive fertility-sparing surgery for cervical cancer is gaining increasing interest, both to achieve a cure and for future fertility. Procedures for robotic radical trachelectomy involving uterine reconstruction are not fully established.

**Methods:**

This study prospectively verified the feasibility and safety of robotic radical trachelectomy between February 2018 and May 2022. The criteria were almost identical to those for our standard abdominal radical trachelectomy. Larger tumors (> 2 cm in diameter) were acceptable for surgery, provided a secure ≥ 1 cm cancer-free space was identified between the tumor and internal os.

**Results:**

Eight patients (median age, 32 y) were registered; the median body mass index was 21.8, and the median tumor size was 11.5 mm (range 0–30 mm). Robotic radical trachelectomy could be achieved in all patients with hybrid sentinel lymph node navigation surgery, confirming the precise cervical amputation line with a newer small knob ultrasonography probe, adequate cervical cerclage with non-absorbable monofilament stitches, and avoiding looseness between vaginal–uterine anastomosis with uninterrupted barbed U-shaped sutures. None of the cases were converted to laparotomy or radical hysterectomy, and there were no major complications. The median follow-up period was 49.5 mo (range 21–58 mo) and no patient had disease recurrence.

**Conclusion:**

Robotic radical trachelectomy is safe and feasible using newer technologies without reducing radicality; it is also less invasive. Procedures are consistently reproducible and have the potential to be generalized to minimally invasive approaches.

## Introduction

The importance and popularity of fertility-sparing surgery (FSS) is increasing annually because of the growing trend of delaying childbearing [1]. New cases of cervical cancer have been reported in patients in their thirties and forties, and this matches the peak in the age distribution of the number of births in Japan [2]. In patients aged ≤ 39 years, Stage I accounted for 79% and Stage II accounted for 14%, showing that younger patients tend to be in earlier stages [3].

In 1987, Dargent et al. [4] first examined laparoscopic vaginal radical trachelectomy (VRT), which is a combination of vaginal modified radical hysterectomy and laparoscopic lymphadenectomy, as FSS. Abdominal radical trachelectomy (ART) was launched based on abdominal radical hysterectomy (ARH) to achieve more flexible removal of the cardinal ligament and appropriate cervical cerclage [5, 6].

The authors have performed more than 265 cases of ART since 2005, with only five cases (2.2%) of recurrence and two cases (1.3%) of death [7]. The overall pregnancy rate among patients who then attempted to conceive was 41% [7]. In addition to curing cervical cancer, trachelectomy aims to deliver successful fertility and pregnancy outcomes. Recent data show a higher pregnancy rate in patients undergoing minimally invasive radical trachelectomy (RT) than ART [8].

Although the next developments in FSS that included robotic minimally invasive surgery (MIS) were proposed in 2008 [9–11], there has been limited research evaluating robotic radical trachelectomy (RRT) [6, 12–20]. RRT provides some advantages, including improved stability in abdominal wounds, shorter hospital stay, less estimated blood loss, and fewer complications than ART [15]. However, a recent systematic review reported that 20% of patients with Stage 1B1 disease had close or positive margins when treated with RRT [21]. Standard procedures for RRT have not yet been established.

We considered four major problems that must be overcome in RRT to consistently achieve outcomes in terms of both radicality and fertility, as follows: (1) detection of a sentinel lymph node (SLN) during robotic surgery, (2) identification of the cervical amputation line, () handling a needle to perform cervical cerclage, and (4) vaginal-uterine anastomosis. Based on these findings, we prospectively evaluated the feasibility and safety of RRT for cervical cancer treatment.

## Materials and methods

### Study design

This study was a prospective, single-arm, phase II trial, and a specific clinical trial in the Clinical Trials Act of Japan; it aimed to verify the feasibility, safety, and treatment outcomes of RRT performed with the Da Vinci Xi surgical system (Intuitive Surgical Inc., Sunnyvale, CA, USA) at Kagoshima University Hospital for fertility-sparing patients with early cervical cancer. The patients were recruited between February 2018 and May 2022. The primary outcome was the surgical completion rate of robotic trachelectomy. Secondary outcomes were blood loss, operative time, complications intra- and post-surgery, length of hospital stay, accuracies of preoperative diagnoses, conversions to laparotomy, pregnancy outcomes, and survival.

The inclusion and exclusion criteria in our study are presented in Table 1. The authors allowed the inclusion of larger tumors [> 2 cm tumor diameter (TD)] under the stipulation of providing a secure ≥ 1 cm cancer-free space between the tumor’s edge and internal os during preoperative imaging. Patients with a TD of > 3 cm underwent pelvic lymph node dissection.

**Table 1:** Inclusion & exclusion criteria strategy for robotic trachelectomy in cervical cancer

Preoperative inclusion criteria:
HSIL/AIS to International Federation of Gynecology and Obstetrics (FIGO, 2008) stage 1B1 cervical cancer with ≤ 1 cm cancer-free space between tumor’s edge and the internal OS in imaging
1) 20 to 45 years old patients who desire fertility sparing surgery and have no evidence of infertility
2) No evidence of distant metastasis, lymph node metastasis, extrauterine invasion in imaging,
3) Simple trachelectomy: HSIL/AIS or stage 1A1 cervical cancer which was not suitable for follow-up after cervical conization
4) Modified radical trachelectomy (including lymph node sampling/dissection): i) In SCC, FIGO stage IA2 or stage IB1 with ≤ 2 cm wider, ii) In adenocarcinoma, FIGO stage IA2 or stage IB1 with ≤ 1 cm wider exophytic tumors
5) Radical trachelectomy (including lymph node sampling/dissection): i) In SCC, FIGO stage IB1, ii) In adenocarcinoma, FIGO stage IB1 with ≤ 3 cm wider
Preoperative exclusion criteria:
1) Simultaneous or metachronous double cancers
2) Special types of adenocarcinoma
3) Untreated cerebral aneurysm
4) Mental disorder
5) Glaucoma
Intraoperative inclusion criteria:
1) No lymph node metastasis detected by the sentinel lymph node biopsy
2) A ≥ 5 mm cancer-free margin in the cervical canal of the extirpated cervix

*HSIL* high-grade intraepithelial lesions, *AIS* adenocarcinoma in situ, *SCC* squamous cell carcinoma

Complications related to surgery were graded using the Clavien–Dindo Classification v.2.0. All surgeries were performed by a gynecologic oncologist with a Class A Robo Doc Certificate from the Japan Robotic Surgery Society. This trial was conducted in accordance with the Declaration of Helsinki, and the protocol was approved by the Institutional Review Board (18-K42) of our institute before patient enrollment. This study was commissioned by Intuitive Surgical Inc. to determine the feasibility of RRT in Japan. Although there is a conflict of interest between the researcher and Intuitive Surgical Inc., this study was planned independently by the researcher, and the company was not involved in the planning, implementation, analysis, and reporting; therefore, there was no effect on the interpretation of results.

### Robotic radical trachelectomy surgery procedures: our standard protocols

Contrast computed tomography (CT) and contrast magnetic resonance imaging (MRI) were performed during preoperative examinations to judge patient qualification for inclusion in the study. The day prior to surgery, 99 mTc-labeled phytic acid as a tracer (RI method) was injected just under the normal mucosa on the outer side of the uterine cervical lesion for the intraoperative SLN biopsy, followed by lymphoscintigraphy and SPECT-CT imaging. SPECT-CT was performed for up to one hour from RI injection to sentinel identification. Intraocular measurements were commonly performed preoperatively to prevent intraoperative elevation of intraocular pressure in patients with occlusive glaucoma due to the prolonged head-down positioning.

#### Procedure steps

##### Producing the vaginal cuff

Prior to the surgical procedure, indocyanine green (ICG method) diluted tenfold was injected around the cervical region at the 3 and 9 o’clock positions. Following the administration of saline solution containing epinephrine beneath the vaginal mucosa, the vaginal wall was transected at a distance of 2 cm from the cervix, and the anterior–posterior wall was ligated to prevent cervical exposure, which can lead to tumor migration.

##### Docking toward the lower abdomen

Patients were placed in the Trendelenburg position (> 20°) adjusted for adequate exposure before docking. Antiskid Pink Pads® were used between the bed and the patient’s back to avoid movement. Port placement remained consistent across surgical procedures in our institute, as described previously [22]. The endoscope port was introduced at the midline at the level of the umbilicus, and other ports were placed laterally, aligned at the level of the endoscope port. Three robotic ports and an assistant port (12 mm) were placed laterally at 6–10 cm according to the habitus or the internal anatomy: the 2nd arm was docked to the endoscope port; the 1st arm to the left, and the 3rd and 4th arms to the right of the endoscope port; lastly, an assistant port (12 mm) was docked to the initial endoscope port.

##### Sentinel lymph node biopsy

A scan of the pelvic lymph node region was performed with a gamma probe to identify and remove the SLN after developing the retroperitoneum. If no metastases to the SLN were seen, based on the results of an intraoperative frozen section, the surgery proceeded as planned. For SLN detection, a laparoscopic-type wireless gamma probe was used to identify hot nodes (RI method) through the assistant port. Concurrently, a near-infrared light camera, which was already fitted with the Da Vinci Xi, named ‘Firefly System®’ (Intuitive Surgical Inc., Sunnyvale, CA, USA) was used to detect a ‘bright node.’

##### Uterine artery transection and cardinal ligament dissection

The uterine artery was incised up to its intersection with the ureter to reduce increased intraoperative bleeding and to reduce the formation of aneurysms during pregnancy. The cardinal ligament and paracolpium were incised on the pelvic side (Piver III) and ligated. Scooping was performed at a level exceeding that of the pelvic nerve plexus.

##### Rectovaginal ligament dissection, and vesico-uterine ligament anterior sheath/posterior sheath dissection

The Douglas fossa peritoneum was incised, and the rectovaginal connective tissue was dissected using a vessel sealing system. Dissection of the anterior sheath of the vesicouterine ligament allowed movement of the ureter to the outer side where the blood vessels of the posterior sheath could be identified. After dissecting the posterior leaf, only the exposed blood vessels were excised to preserve the bladder nerve branch near the vaginal canal.

##### Cervical cerclage and vaginal canal/cervical canal amputation

**Fig. 1:**
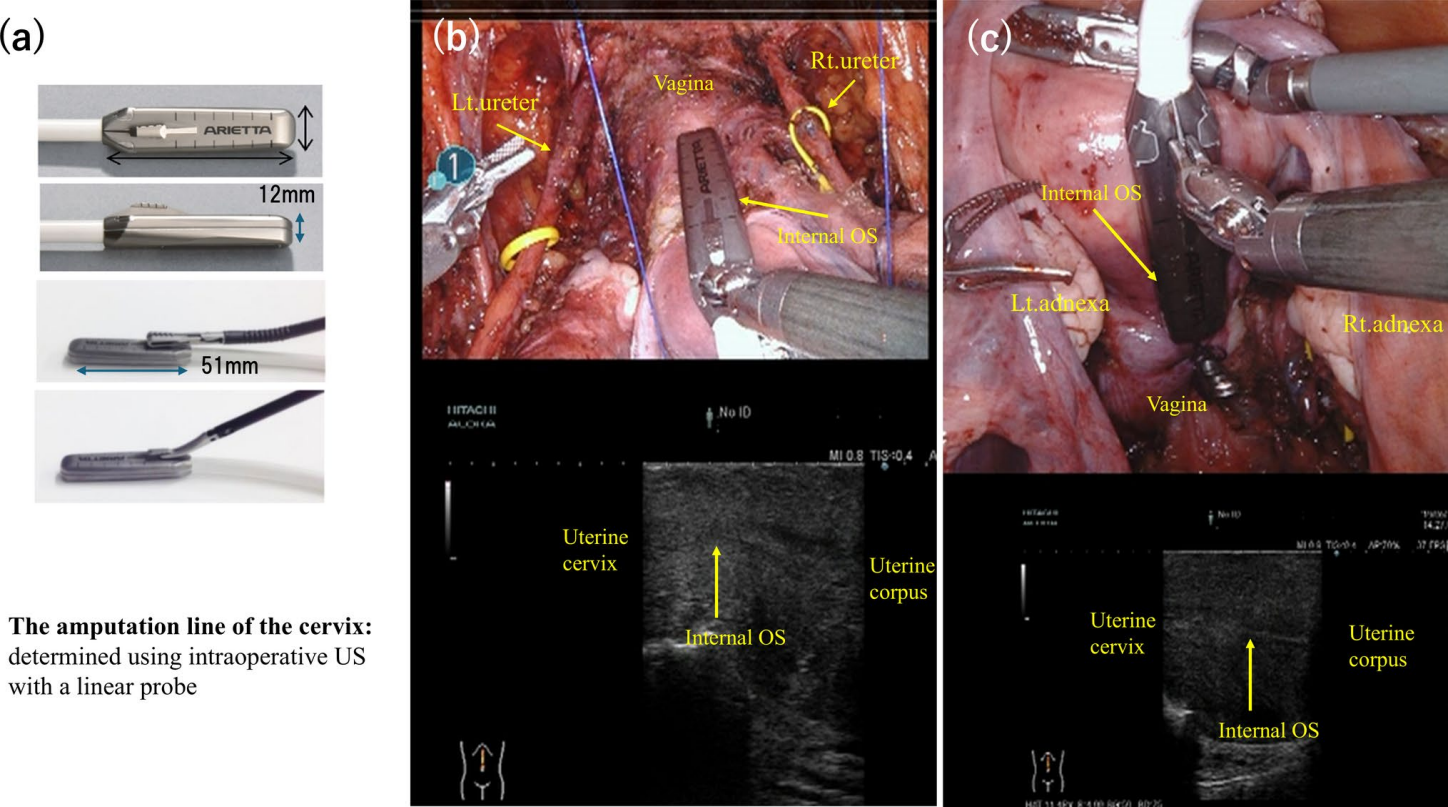
The site of amputation was determined by intraoperative ultrasonography using a small linear probe (**a**). Identification of the internal os in the ventral (**b**) and dorsal regions (**c**)

The amputation line of the cervix was determined using intraoperative US with a linear probe L43K® (Hitachi Aloka Co. Ltd., Tokyo, Japan) (Fig. 1a). First, a probe that could pass through the 12 mm assist port was placed directly on the surface of the uterine cervical wall, and the position of the endocervical canal was confirmed. Incision marks were drawn such that the cervical canal remained at least 5–10 mm from the endocervix, to the vaginal side. The Robotic EndoWrist (Intuitive Surgical Inc., Sunnyvale, CA, USA) could easily grasp the probe, and we were able to detect the precise line of the internal os through the anterior and posterior uterine surfaces while viewing on a multiple display system (Fig. 1b, c).

**Fig. 2:**
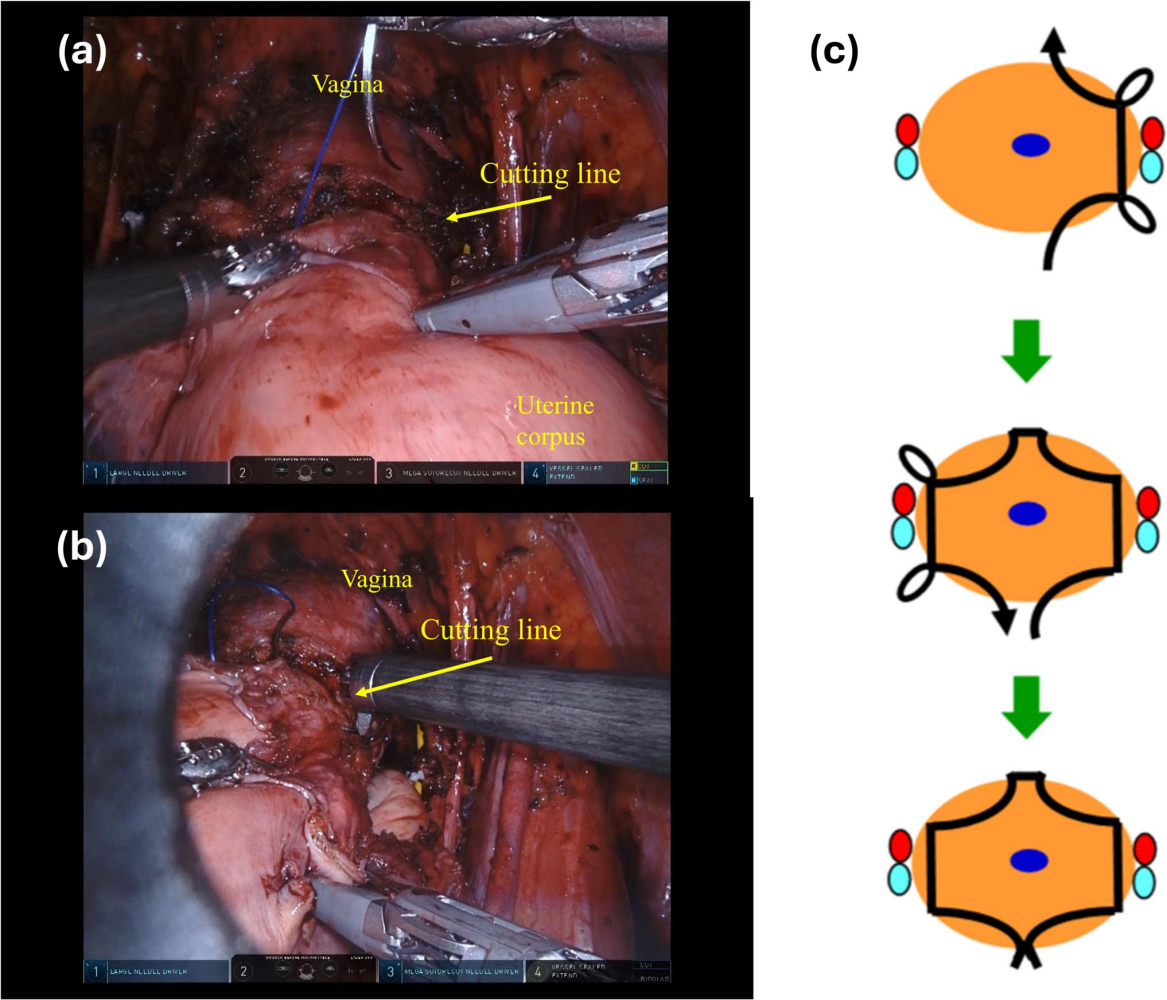
Intraoperative photographs of cervical cerclage (**a**, **b**), and schematic of stitches (**c**). Cerclage was performed using non-absorbable monofilament stitches, similar to the McDonald’s method

Cervical cerclage was performed using No. 1 Prolene ® (non-absorbable monofilament stitch) (Ethicon GmbH., Norderstedt, Germany), as per McDonald’s method (Fig. 2). A double cervical suture was performed before the cervical canal was amputated because, thereafter, the uterus moves and makes it difficult to perform the stitching. Cerclage should not be too tight, and to check this, an intrauterine device (IUDs) was inserted from the neocervix without any problems after vaginal suturing.

The cervical canal was amputated using a monopolar electrode, whereas the cervical gland lesion was dissected without cauterization. The resected cervix was sent to be frozen; trachelectomy was only performed if carcinoma was not present within 5 mm of the cut edge or the lesion, and no additional resection was allowed. We preserved the uterus in situ only when there was sufficient confirmation of a cancer-free margin from the amputated edge. Robotic radical hysterectomy was performed when there were no secure cancer-free cervical margins.

##### Anastomosis of the neocervix and vaginal stump

**Fig.3:**
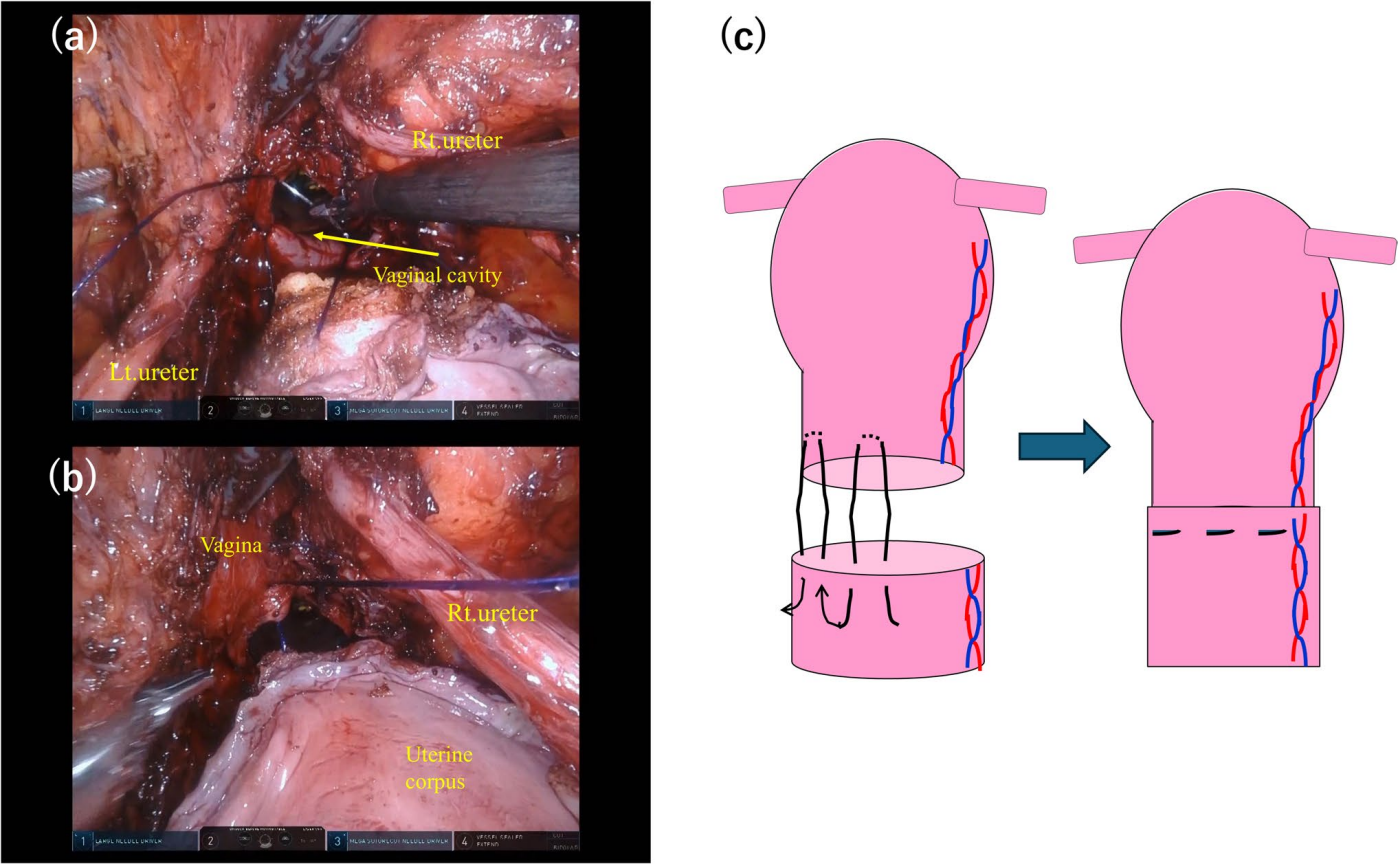
Intraoperative photographs of anastomosis of the neocervix and vaginal stump (**a**, **b**), and schematic of a continuous U-shaped suture (**c**). Anastomosis of the neocervix and vaginal stump was performed using uninterrupted U-shaped sutures with a barbed monofilament stitch, which covered the neocervix with the vaginal wall

Uninterrupted U-shaped sutures using STRATAFIX® (STRATAFIX Spiral PDS Plus®) (Ethicon GmbH., Norderstedt, Germany) were stably created to cover the neocervix apposed to the vaginal wall (Fig. 3).

##### Reconstruction of the circular ligament–pelvic peritoneal closure

The peritoneum was closed following drain placement in the retroperitoneal cavity of the pelvis. The round ligament was reconstructed properly. An anti-adhesion agent was applied to the peritoneal suture and around the fallopian tubes.

#### Supplementary information

##### Surgeries that were made difficult through complications such as bleeding, were promptly

Converted to open laparotomy. An IUD was placed immediately after surgery to avoid cervical stenosis. It was removed six months after surgery, following which the patient was permitted to become pregnant. In cases where achieving a natural pregnancy is proving difficult, infertility treatment interventions should be promptly undertaken. Postoperative chemotherapy was administered to patients with intermediate-risk cervical cancer pursuant to postoperative pathological diagnoses according to the Japan Society of Gynecologic Oncology Guidelines. Postoperative outpatient screening is advisable for at least five years following surgery or postoperative chemotherapy.

## Results

The characteristics of the eight patients who underwent robotic trachelectomy for cervical cancer are listed in Table 2. The median age was 32 y (range 21–36 y) and median body mass index (BMI) was 21.8 (range 19.8–30.8). All but one patient were histologically diagnosed with squamous cell carcinoma, and the International Federation of Gynecology and Obstetrics (FIGO) Stage 1B1 was most common. The preoperative estimated median tumor size was 11.5 mm (range 0–30 mm). In Case 2, the tumor was visible on transvaginal examination; however, the tumor was not detected on the preoperative MRI. Only Case 5 underwent prior conization for diagnostic purposes, which resulted in a positive endocervical margin. Table 3 describes the clinical outcomes of the eight patients. The extended surgical time in Case 1 was due to unfamiliarity with the new techniques. SLN were detected in all cases, and none of the patients had lymph node metastasis. All patients who underwent the RI and ICG methods were identical in SLN outcome. Regarding intraoperative frozen sections, all patients had a cancer-free margin at the cervical canal amputation edge and were able to undergo RRT. None of the cases were converted to laparotomy, and neither additional nor unexpected port placement occurred. The photographs in Figs. 1 and 2 show the highlights of Case 4, in which we performed our standard RRT procedures.

**Table 2:** Baseline characteristics of the eight patients with robotic radical trachelectomy

Case no	1	2	3	4	5	6	7	8	Median (range)	Median
Age, years	33	36	31	32	39	30	36	21	32.5 (21–36)	32.5
BMI, kg/m^2^	22.9	21.4	22.2	19.8	30.8	29.6	20	20	21.8 (19.8–30.8)	21.8
History of previous pregnancy	Nil	Nil	Nil	2	2	Nil	Nil	1	2 (1–2)	2
Prior parity	Nil	Nil	Nil	1	1	Nil	Nil	1	1	1
Prior conization	Nil	Nil	Nil	Nil	Yes	Nil	Nil	Nil	n/a	n/a
Prior laparotomy	Nil	Nil	Nil	Nil	Nil	Nil	Nil	Nil	n/a	n/a
Histologic type	SCC	SCC	SCC	SCC	SCC	SCC	Mucinous carcinoma	SCC	n/a	n/a
Tumor size, mm	20	5	30	28	11	12	0	10	11.5 (0–30)	11.5
*FIGO stage	I B1	I B1	I B1	I B1	I B1	I B1	1B1	1A1	n/a	n/a
Follow-up, month	48	57	58	56	50	49	33	21	49.5 (21–58)	49.5

* BMI* body mass index, *n/a* not applicable, *SCC* squamous cell carcinoma, *FIGO* International Federation of Gynecology and Obstetrics

^*^Tumor size was measured by resected specimen

**Table 3:** Clinical outcomes and complications in robotic radical trachelectomy

Surgical outcome	1	2	3	4	5	6	7	8	Median (range)
Blood loss, ml	895	65	65	75	65	71	15	125	68 (15–895)
Operation times, minutes	741	474	422	466	380	491	367	380	444 (367–741)
Console times, minutes	635	377	320	368	294	329	295	310	324.5 (295–635)
Surgery	RRT + SNNS	RRT + SNNS	RRT + SNNS	RRT + SNNS	Robotic MRT + SNNS	Robotic MRT + SNNS	Robotic MRT + SNNS	Robotic MRT + SNNS	N/A
Detection of SLN	Yes	Yes	Yes	Yes	Yes	Yes	Yes	Yes	N/A
Location of SLN									N/A
Right	Int ilic LN	Obturator LN	Obturator LN	Obturator LN and Ext ilic LN	Obturator LN	Obturator LN	Obturator LN	Obturator LN	N/A
Left	Ext ilic LN	Obturator LN	Obturator LN and Ext ilic LN	Obturator LN	Obturator LN	Obturator LN	Obturator LN	Outer iliac LN	N/A
Nodal metastases	Nil	Nil	Nil	Nil	Nil	Nil	Nil	Nil	N/A
Length of hospital stay, days	10	8	8	10	7	7	7	7	7.5 (7–10)
Intraoperative complications	Nil	Nil	Nil	Nil	Nil	Nil	Nil	Nil	
Postoperative complications	Extremities pain, Grade 1	Residual urine, Grade 1	Residual urine, Grade 1	Sub ileus; pelvic infection, grade 2	Nil	Nil	Residual urine, Grade 1	Residual urine, Grade 1	
Obstetrical outcomes									
Attempting conception	Yes	Yes	Yes	Nil	Yes	Nil	Yes	Yes	
Attempting conception with ART	Nil	Yes	Nil	Nil	Yes	Nil	Nil	Yes	
Abortion	Nil	20 weeks of pregnancy, CAM susp	Nil	Nil	once	Nil	Nil	Nil	
Live birth	Nil	Nil	Nil	Nil	Yes, 36 weeks of pregnancy complicated with total previa	Nil	Nil	Nil	

* RRT* robotic radical trachelectomy, *MRT* modified radical trachelectomy, *SNNS* sentinel node navigation surgery, *N/A* not applicable, *SLN* sentinel node, *LN* lymph node, *ART* assisted reproductive technique, *CAM* chorioamnionitis

No major grade 3 intraoperative or postoperative complications occurred. Regarding postoperative complications, Case 1 reported grade 1 extremity pain; in Case 4, grade 2 pelvic infection was diagnosed by the appearance of a fever on postoperative day 15 and the patient recovered after receiving intravenous antibiotics without requiring surgical intervention. The median follow-up period was 49.5 mo (range 21–58 mo), and none of the patients showed signs of disease recurrence. No patients had risk factors in the final pathological diagnoses that required postoperative chemotherapy.

Regarding obstetrical outcomes, two patients had menstrual irregularities, and two out of six patients who attempted to conceive became pregnant; of these, one was aborted at 20 weeks of pregnancy and the other was complicated with total previa and delivered by cesarean section at 36 weeks of pregnancy.

## Discussion

All patients included in this study underwent successful robotic trachelectomy with no major complications. The RRT criteria were almost identical to our standard ART criteria [23], and all patients completed robotic trachelectomy without being converted to ART or RH. No serious grade 3 complications occurred during the perioperative period.

We performed RRT using similar procedures to ART because the surgical outcome of Piver III of ART is considered to be superior for larger tumors of Stage IB1 with 2 cm < TD compared to VRT [8, 24], and also based on our positive results of oncological outcomes in a previous ART study [25].

The four major problems mentioned above are discussed below. The application of sentinel lymph node navigation surgery (SNNS) for patients with Stage IA-IB1 disease with smaller tumors (TD ≤ 3 cm) in cervical cancer has been established [26, 27]. In ART, we have concurrently used the RI and ICG methods as double tracers to accurately detect SLN. This hybrid method could be achieved by combining a laparoscopic type-wireless gamma probe, and ‘Firefly System®’, fitted with the Da Vinci Xi. SLN could be detected in all our patients, which assisted progress in trachelectomy procedures.

Determining the cervical amputation line is the most difficult and essential objective of robotic trachelectomies. Although most studies report the surgical procedures in RRT, we could not find reports describing the avoidance of inaccurate lines of cervical amputation. We considered employing the same ultrasonography (US) used in our ART procedure as the best way to precisely detect the amputation line for RRT. The existing US was too large to enter through the port, so we first introduced a newer small knob US probe and were able to confirm the precise line of the internal os while concurrently viewing a multiple display mode named ‘Tile Pro system®’ (Intuitive Surgical Inc., Sunnyvale, CA, USA).

We performed prophylactic cervical cerclage after cervical amputation in ART because uteri were consistently fixed by grasping them directly with the fingers, whereas we confirmed cerclage before cervical amputation when performing RRT. This decision was made because cerclage using the robotic EndoWrist® for a mobile unstable uterus is difficult after cervical amputation. We also used Prolene® for cerclage, as recommended in a previous report that compared several sutures [6]; the use of Prolene® did not lead to cervical stenosis or signs of infection.

Regarding vaginal–uterine anastomosis, covering the neocervix with the vaginal wall is important to avoid vaginal stenosis and decreased uterine mobility, which can lead to postoperative coital pain. Although we used multiple interrupted U-shaped sutures in ART, this type of suture was difficult to perform using the robotic EndoWrist®. Therefore, we used uninterrupted sutures with the STRATAFIX Spiral®, which has many barbs to avoid looseness during continuous suturing; the complete length is 15 cm and is suitable for handling with a robotic EndoWrist®. Using this suture, the external os of the neocervix can be placed in the center of the vagina.

These procedures for achieving robotic trachelectomy might lead to the development of newer devices and further improvements in robotic systems that allow for an increase in the range of motion and distance. No studies to date have consistently recreated ART procedures in RRT; it is possible that they may not be achievable without the Da Vinci Xi system. However, we could not find a report evaluating RRT using the latest Da Vinci Xi system.

In the USA, the proportion of MIS for trachelectomy has rapidly increased from 29.3% in 2010 to 75% in 2015, and 66.6% of patients undergoing MIS have undergone robotic surgery [28]. In Matsuo et al. [26], the survival rates were almost identical (95.7% in MIS vs. 92.3% in laparotomy) to the current study. Although no serious complications have occurred in our cases, common complications regarding trachelectomy by MIS include cervical stenosis (0–7.1%), cervical erosion (0–11.9%), amenorrhea (13%), and intra-abdominal abscess or peritonitis [15, 29]. However, available evidence regarding the feasibility or safety of robotic trachelectomy is limited compared to that of the pure laparoscopic procedure [21, 28]. Considering the results of LACC trials, we should be concerned about these outcomes in patients undergoing RRT [20, 28, 30].

Since FFS was initiated by Dargent et al. [4], efforts to tailor techniques, referring to tumor-associated prognostic factors and optimizing reproductive potential, are the main trends. Fertility results comparing FSS procedures have recently been reported [8]. A significant difference in fertility rates has been noted between each FSS procedure: 44% in ART, 57% in VRT, and 65% in minimally invasive RT, respectively. In our previous report on ART and abdominal modified radical hysterectomy (AMRT), the overall pregnancy rates were 34% and 50%, respectively. The median interval between trachelectomy and initial post-trachelectomy pregnancy was 38 months, and 79% of pregnancies were achieved after infertility treatment [7]. In addition to adhesion formation [31], these higher rates of fertility and premature delivery are attributed to cervical factors, such as cervical mucus production, cervical stenosis, and shortened cervix [25, 32]. Many fetal losses and premature deliveries are related to premature rupture of membranes. A cervical length < 10 mm may lead to a decrease in pregnancy rates [33]. However, a greater length of the preserved cervix is related to the intraoperative conversion rate (i.e., fertility loss rate) [25]. Therefore, we carefully determined the extent of cervical preservation. Although our study of obstetric outcomes had insufficient data for analysis, our accurate line decision for cervical amputation by US has the potential to become a leading positive factor for obstetric outcomes considering the future tailored treatment strategy in patients who desire FSS treatment.

Regarding tumor diameter in the inclusion criteria, our institute performs ART and AMRT using the same laparotomy technique, and we have adopted the AMRT approach of TD ≤ 2 cm based on the results of a prospective study that showed that ≤ 2 cm is feasible [34]. The upper limit of tumor size for SCC was set at 4 cm, as our series of procedures have been shown to be well tolerated, and easily converted to hysterectomy in high-risk cases, and with reference to other reports [35, 36]. Although few studies exist regarding the effectiveness of postoperative chemotherapy for trachelectomy or fertility, the recurrence rate for preoperative chemotherapy of tumors > 2 cm in diameter was only 9.2%, and 78% of fertility was preserved [37, 38].

The number of patients in this study was the minimum required for verification of the main purpose of the study; in addition, relatively long-term follow-up of oncological and obstetrical outcomes were also determined. Incorporation of MIS approaches is the main component of MIS development, which contributes to minimizing the morbidity of trachelectomy [30]. A literature review of the original report regarding the RT, following a PubMed search using the keywords with “robotic” and “trachelectomy,” is shown in Table 4 [6, 14–16, 20, 39]. Reports with insufficient data were excluded [29, 40–42]. Evidence from Asia, particularly Japan, has not yet been reported. Previous reports have not clearly resolved the suggested four important problems: meanwhile, our procedures have been consistently reproducible and have the potential to become standard RT protocols.

**Table 4:** The literatures of the robotic trachelectomy for fertility-sparing cervical cancer

Authors	Year (y)	Trial	Robotic System	Age (median, y)	stage,size (median) (mm)	Number of patients	BMI (Median)	Operation times (min, Median)	Blood loss (ml, Median)	Number of transfusion patients	Uterine artery preservation	Cerclage	Number of conversions	Perioperative complications	Post-operative major complications	Recurrences(n)	Number of Attempting pregnancy	Number of pregnancies	Pregnancy outcomes	Follow-up (months)
Ramirez et al. [20]	2010	Retrospective	Da Vinci	32	1A1 to 1A2	4	27.1	339.5	62.5	Nil	Nil	Yes	Nil	Nil	A patient with transient left lower extremity sensory neuropathy was identified. This condition persisted for about 20 days and ultimately resolved spontaneously	Nil	Nil	Nil	Nil	3 (105 days)
Nick et al. [15]	2012	Retrospective	Unknown	29.8	1A to 1B1,21.5 mm (mean)	12	Unknown	294 (207–379)	62.5 (25–450)	Nil	Nil	Yes	4 (all patients converted to radical hysterectomy)	Two fever, one urinary tract infection, one irregular menstrual bleeding or amenorrhea	Although there was no significant difference in the incidence of individual health problems between surgical techniques, patients who underwent open radical trachelectomy experienced a higher cumulative rate of late health issues compared to those who underwent robotic radical trachelectomy (58% vs. 13%, respectively; p = 0.07)	Nil	unknown	Nil	Nil	10.8 months (0.43–24.6)
Persson et al. [16]	2012	Prospective	da Vinci S or Si		1A to 1B1	13		297(mean)	133(mean)	N/A		Yes	Nil	Nil	Nil	Nil	5	4	Four women have spontaneously become pregnant, with one delivering in the 35th week of pregnancy and becoming pregnant again, another delivering in the 36th week, and the remaining two having their pregnancies ongoing	Nil
Johansen et al. [6]	2016	Prospective	Unknown	30(23–41)	1A to 1B1	56		321	75 (0–300)	Nil	Yes	Yes	7	Nil	One vesicovaginal fistula and one compartment syndrome. The former was treated with conservative management, while the latter underwent a decompressive fasciotomy	4	21	15	A total of 20 pregnancies were documented, including one first trimester miscarriage, two ongoing pregnancies, and 17 completed pregnancies that resulted in 18 children (one of which was a twin pregnancy). Out of the 20 pregnancies, only five women (71%) were delivered via elective Cesarean section between GW 36–38. None of the women gave birth naturally	27 (1–89)
Hong et al. [14]	2011	Retrospective	Da Vinci	Unknown	1B1	3	Unknown	275 (console time)	23 (mean)	None	Yes	Yes	Nil	One aorta bleeding, one active bleeding	None	nil	unknown	Nil	Nil	8 (6–9)
Ekdahl et al. [32]	2022	Prospective	Unknown	31(18–42)*	1A1with LVSI to 2A,9 (3–20)*	166*	23.5 (17.0–47.0)*	Unknown	Unknown	Unknown	all but five	Yes	6	Bladder injury (n = 1) Compartment syndrome of the leg (n = 1)	Early complications (< 30 days) Clavien–Dindo IIIa-IIIb n = 10 (6.7%) Vesico-vaginal fistula (n = 1) Pelvic lymph seroma (n = 4) Pelvic hematoma (n = 2) Bowel obstruction (n = 1) Vaginal bleeding (n = 2) Late complicationsa (> 30 days) n = 11 (7.4%) Persistent vaginal bleeding (n = 6) Voiding problems (n = 4) Vesico-vaginal fistula (n = 1) Cervical stenosis n = 18b	9	88	103	Preterm delivery n = 18 GW < 28 + 0 n = 3 GW 28 + 0 until 31 + 6 n = 4 GW 32 + 0 until 33 + 6 n = 1 GW 34 + 0 until 36 + 0 n = 10 Preterm delivery for indications likely unrelated to RRT n = 4 preeclampsia n = 3, placenta previa n = 1	64 (2–140)*
Kobayashi et al. (present study)	2024	Prospective	da Vinci Xi	32.5 (21–36)	1A to 1B1, 11.5 (0–30)	8	21.8 (19.8–30.8)	444 (367–741)	68 (15–895)	Nil	Nil	Yes	1	Nil	Extremities pain (n = 1), subileus (n = 1), pelvic infection (n = 1)	Nil	8	2	In two pregnant patients, one was aborted at 20 weeks of pregnancy, and the other was complicated with total previa and delivered by cesarean section at 36 weeks of pregnancy	49.5 (21–58)

*BMI* body mass index, *N/A* not applicable, *LVSI* lymphovascular space invasion

^*^Include the patients with fertility could not be preserved

In conclusion, we demonstrated that RRT for cervical cancer could be performed in a safe and feasible manner using updated technologies. Furthermore, RRT can be used without reducing radicality and is less invasive. Therefore, we predict a rapid increase in the selection of trachelectomy in MIS. Nevertheless, MIS treatment strategies in fertility-sparing patients with cervical cancer that consider the balance between radicality and fertility, including preoperative evaluation, inclusion criteria, and adequate surgical procedures, should be addressed in future studies.

## Data Availability

The data used in this study are available upon reasonable request by writing to the corresponding author.
